# Parietin Cyclodextrin-Inclusion Complex as an Effective Formulation for Bacterial Photoinactivation

**DOI:** 10.3390/pharmaceutics14020357

**Published:** 2022-02-04

**Authors:** Abdallah Mohamed Ayoub, Bernd Gutberlet, Eduard Preis, Ahmed Mohamed Abdelsalam, Alice Abu Dayyih, Ayat Abdelkader, Amir Balash, Jens Schäfer, Udo Bakowsky

**Affiliations:** 1 Department of Pharmaceutics and Biopharmaceutics, University of Marburg, Robert-Koch-Str. 4, 35037 Marburg, Germany; abdallah.ayoub@pharmazie.uni-marburg.de (A.M.A.); bernd.gutberlet@pharmazie.uni-marburg.de (B.G.); eduard.preis@pharmazie.uni-marburg.de (E.P.); ahmed.abdelsalam@pharmazie.uni-marburg.de (A.M.A.); alice.abudayyih@pharmazie.uni-marburg.de (A.A.D.); aboelela@staff.uni-marburg.de (A.A.); j.schaefer@staff.uni-marburg.de (J.S.); 2 Department of Pharmaceutics and Industrial Pharmacy, Faculty of Pharmacy, Zagazig University, Zagazig 44519, Egypt; 3 Department of Pharmaceutics and Industrial Pharmacy, Faculty of Pharmacy, Al-Azhar University, Assiut 71524, Egypt; 4 Assiut International Center of Nanomedicine, Al-Rajhy Liver Hospital, Assiut University, Assiut 71515, Egypt; 5 Department of Pharmaceutical Chemistry, University of Marburg, Marbacher Weg 10, 35032 Marburg, Germany; amir.balash@pharmazie.uni-marburg.de

**Keywords:** physcion, hydroxypropyl-β-cyclodextrin, photosensitizer, antimicrobial photodynamic therapy

## Abstract

Multidrug resistance in pathogenic bacteria has become a significant public health concern. As an alternative therapeutic option, antimicrobial photodynamic therapy (aPDT) can successfully eradicate antibiotic-resistant bacteria with a lower probability of developing resistance or systemic toxicity commonly associated with the standard antibiotic treatment. Parietin (PTN), also termed physcion, a natural anthraquinone, is a promising photosensitizer somewhat underrepresented in aPDT because of its poor water solubility and potential to aggregate in the biological environment. This study investigated whether the complexation of PTN with (2-hydroxypropyl)-β-cyclodextrin (HP-β-CD) could increase its solubility, enhance its photophysical properties, and improve its phototoxicity against bacteria. At first, the solubilization behavior and complexation constant of the PTN/HP-β-CD inclusion complexes were evaluated by the phase solubility method. Then, the formation and physicochemical properties of PTN/HP-β-CD complexes were analyzed and confirmed in various ways. At the same time, the photodynamic activity was assessed by the uric acid method. The blue light-mediated photodegradation of PTN in its free and complexed forms were compared. Complexation of PTN increased the aqueous solubility 28-fold and the photostability compared to free PTN. PTN/HP-β-CD complexes reduce the bacterial viability of *Staphylococcus saprophyticus* and *Escherichia coli* by > 4.8 log and > 1.0 log after irradiation, respectively. Overall, the low solubility, aggregation potential, and photoinstability of PTN were overcome by its complexation in HP-β-CD, potentially opening up new opportunities for treating infections caused by multidrug-resistant bacteria.

## 1. Introduction

The overuse of antibiotics has resulted in inexorable antibiotic resistance, which worsens day by day, extending hospital admissions, raising healthcare costs, and eventually leading to increased death rates. In 2020, more than 670,000 people were infected in Europe with antibiotic-resistant bacteria, with approximately 33,000 deaths. The total economic burden of antibiotic resistance in Europe is expected to be 1.1 billion Euros [1]. The loss in economic output due to illness or death-related infection was estimated to surpass 1 trillion USD per year after 2030 and approach 2 trillion USD annually by 2050, accounting for a total of 100 trillion USD decline in global production between 2014 and 2050 [2]. Even worse, the number of deaths caused by worldwide antibiotic resistance is expected to increase to 10 million per year by 2050, up from 700,000 in 2014 [3]. Development of antibiotic resistance results in higher costs because of switching to more expensive antibiotics [4]. Multiple reasons, scientific, economic, and regulatory, hinder the development of new antibiotics [5]. Moreover, other drugs for chronic diseases are more appealing to pharmaceutical companies than short-course antibiotics. Consequently, approvals of new systemic antibiotics by the US Food and Drug Administration have dropped by 90% in the last 30 years [5]. Thus, alternative treatment strategies against resistant bacteria are urgently sought.

Photodynamic therapy (PDT) against cancer cells or antimicrobial photodynamic therapy (aPDT) have advantages over traditional treatments, e.g., minimal invasiveness and excellent safety profiles [6]. PDT and aPDT for selective damage to the area of interest depend on three nontoxic components: a photosensitizer (PS), light, and oxygen [7]. They are two-step procedures in which a photosensitizer (PS) is administered to patients, followed by light irradiation at a specific wavelength matching the absorbance spectra of the PS. Once exposed to light, the PS is excited from its low-energy ground state into a high-energy singlet state which undergoes intersystem crossing to the longer-lived triplet state [6]. Via type I and type II mechanisms, the PS in the triplet state can generate reactive oxygen species (ROS) such as singlet oxygen superoxide anions and hydroxyl radicals, which are the leading cause of cell death [8].

Unlike traditional antibiotics, which have a particular target known as the key-hole principle, this mechanism singles out aPDT as a multi-target procedure leaving no room for resistance development [9]. Additionally, aPDT does not necessitate a specific extracellular or intracellular localization of the PS to exert its damaging effect [9]. As a result, bacteria are unlikely to establish antibiotic resistance against this multi-targeted approach even after repeated administration [8]. Furthermore, such a broad-spectrum activity against gram-positive and gram-negative bacteria, fungi, viruses, and protozoa is beneficial in the empirical therapy of undiagnosed infections. Another advantage of aPDT is that fast-growing cells such as bacteria can accumulate PSs at a higher rate, resulting in increased selectivity [10]. This is especially harmful to bacteria, as their DNA is not isolated from the cytoplasm like that of eukaryotic cells [11]. Additionally, the photodestructive effect of aPDT can be physically controlled in practice by local light applications. The photoantimicrobial effect is much faster than traditional therapies that can take days to be effective [12]. Moreover, the generated ROS during PDT can chemically oxidize the virulence factors such as lipopolysaccharide, protein toxins, proteases, and α-hemolysin [11]. Although extensive work has been performed in aPDT, only three photosensitizers (methylene blue, toluidine blue O, and indocyanine green) have received clinical approval in dentistry so far [12].

Parietin (PTN), also termed physcion, is an anthraquinone naturally present as a secondary metabolite in lichens (e.g., *Xanthoria parietina* [13]), in other fungi (e.g., *Aspergillus*, *Penicillium* [14]), and also in plants (e.g., *Rheum*, *Rumex*, and *Ventilago* [14]). The UV protectant role of PTN has been detected in lichens adapted to UV stressed environments, whereby the mycobiont synthesizes PTN to protect the photobiont against oxidation mediated by excessive solar radiation [14,15]. *Xanthoria parietina* has been used in traditional medicine for kidney problems and menstrual disorders and as a painkiller [16]. Although PTN offers several activities, including antibacterial and antifungal [14], and has antitumoral [17] and other photosensitizing properties [18,19], it was studied only in organic solvents and is not applicable in clinical use. Like many lipophilic PSs, PTN aggregates unduly in the biological environment, losing its singlet oxygen quantum yield and limiting its routine use. To solve these drawbacks, we recently reported the encapsulation of PTN into liposomes to promote its aqueous solubility and stability in the biological milieu and enhance selectivity and delivery to the targeted cells [19]. However, its high hydrophobicity does not favor entrapping a higher amount in the lipid bilayer. Such a problem can be addressed with cyclodextrin (CD) complexation to increase the inclusion capacity of PTN in the delivery system.

CD is widely utilized as a typical solubilizer for hydrophobic drugs to enhance their aqueous solubility and chemical stability with a high loading capacity and a straightforward procedure [20]. CD can enclose the hydrophobic drug in its central cavity to form a host-guest complex or supramolecular species without altering its framework structure, while the outer surface is still hydrophilic to ensure water solubility [11]. CD is a cyclic biocompatible oligosaccharide structurally composed of 6–8 D-glucopyranose monomers connected by α-1,4-glucose bonds [11]. According to the number of glucose units, the natural CD may differ in water solubility and hydrophobic cone dimension available for drug accommodation. There are different varieties of CD, such as α-CD (six glucose units with a cavity volume of 0.174 nm^3^), β-CD (seven glucose units with a cavity volume of 0.262 nm^3^), or γ-CD (eight glucose units with a cavity volume of 0.427 nm^3^) [21]. However, natural CD, particularly β-CD, can damage the renal tubule either by microcrystalline precipitation in the kidney because of lower water solubility or as a cyclodextrin/cholesterol complex [20]. Therefore, the natural CDs were chemically modified to enhance their aqueous solubility and decrease nephrotoxicity. Among them, hydroxypropyl-β-CD (HP-β-CD) is the most notable derivative, with a greater water solubility of 60% (*w/w*) compared to 2% for its parent form, β-CD [20]. Owing to its biocompatibility and minimal toxicity, HP-β-CD has long been used as a delivery system in PDT for many photosensitizers [11] such as hypericin [10], chlorophyll a [22], aminolevulinic acid [23], temoporfin [24], chlorin e6 [25], and curcumin [26]. These studies showed enhanced water solubility and delivery to the targeted tissues without significantly changing their photophysical properties. To the best of our knowledge, no work has investigated the inclusion of PTN in any cyclodextrin complex.

Various mechanisms of aPDT have already been investigated, but its success depends mainly on the radiation strength and radiant exposure, or the overall dosimetry [27]. Differences among the used photosensitizers require a multifactorial concept in dosimetry that should always be considered [28]. PTN by itself shows a concentration-dependent antimicrobial effect during irradiation and in the dark [17]. Free PTN is not suitable for therapy because of its low water solubility and poor bioavailability, which can be overcome by water-soluble PTN/HP-β-CD complexes. Hegge et al. showed an advantage of their cyclodextrin conjugate in terms of thermal stability, photostability, and easier solubilization than the ethanolic solution [29]. The conjugation to cyclodextrin results in a slightly reduced affinity of photosensitizers for gram-negative bacteria, which is compensated by the improved solubility, availability, and efficiency of singlet oxygen generation [30]. Furthermore, targeting moieties can easily be incorporated into the complex to improve selectivity [31,32].

This study aimed to improve the aqueous solubility of PTN, simultaneously maintaining its photoactivity, to obtain a promising candidate for further use in PDT. The solubility behavior was studied at different concentrations of HP-β-CD. Additionally, the complex formation (PTN/HP-β-CD) was evidenced by various characterizations, such as proton nuclear magnetic resonance (^1^H NMR), Fourier-transform infrared spectroscopy (FT-IR), powder x-ray diffraction (PXRD), scanning electron microscopy (SEM), and differential scanning calorimetry (DSC). PTN delivery with minimal loss of photoactivity was evaluated by the uric acid method assessing the photodynamic activity of PTN/HP-β-CD complexes in water. Furthermore, the photodegradation profile of PTN/HP-β-CD complexes upon exposure to blue LED irradiation was monitored and compared to that of free PTN in ethanol. The photodynamic activity of PTN/HP-β-CD complexes was tested for the first time against gram-positive and gram-negative bacterial strains.

## 2. Materials and Methods

### 2.1. Materials

Parietin (PTN, purity >98%) was purchased from Cayman (Hamburg, Germany). 2-hydroxypropyl-β-cyclodextrin (HP-β-CD, average MW = 1483 g/mol) was obtained from Sigma-Aldrich (Taufkirchen, Germany). Ultrapure water was generated by PURELAB flex 4 (ELGA LabWater, High Wycombe, UK) and used for all experiments in this study. All other chemicals and solvents were of analytical grades and used as received.

### 2.2. Bacterial Strains and Media

Glycerol stock cultures of *Staphylococcus saprophyticus* subsp. *bovis* (*S. saprophyticus*, DSM 18669, DSMZ, Braunschweig, Germany) and *Escherichia coli* DH5 alpha (*E. coli*, DSM 6897, DSMZ, Braunschweig, Germany) were prepared and stored at −80 °C. The stocks were thawed one day before the bacterial viability assay and cultured in Mueller Hinton broth (MHB, Sigma Aldrich Chemie) on an orbital shaker (Compact Shaker KS 15 A, equipped with Incubator Hood TH 15, Edmund Bühler, Bodelshausen, Germany) set at 200 rpm and 37 °C.

### 2.3. Light Source

The irradiation experiment was performed with a prototype low-power LED device consisting of an array of light-emitting diodes custom-made by Lumundus GmbH (Eisenach, Germany). The device can emit light at wavelengths of 457 nm and 652 nm for blue and red regions, respectively. The actual light dose (J/cm^2^) = Irradiance (W/cm^2^) × irradiation time (in sec) [33].

### 2.4. Stoichiometry: Job’s Plot

The continuous variation technique (Job’s plot) was employed to determine the complex stoichiometry [34]. Briefly, equimolar stock solutions of PTN (100 µM in ethanol) and HP-β-CD (100 µM in water) were mixed at different ratios (1:9; 2:8; 3:7, and so on), maintaining a final volume of 10 mL to get different mole fractions of PTN from 0 to 1, while the total concentration of PTN and HP-β-CD remained constant. The suspensions were then stirred overnight on a magnetic stirrer at 200 rpm (IKA RT 15, IKA-Werke, Staufen, Germany). Any insoluble materials were removed by centrifugation of the suspension (16,800× *g* for 15 min) (Centrifuge 5418, Eppendorf, Hamburg, Germany) and filtration of the supernatant (0.45 µm nylon filter, Pall Corporation, New York, NY, USA). The amount of PTN solubilized in the complex was estimated spectrophotometrically at λ = 434 nm and was plotted (Δ*A × R*) against the mole fraction (*R*) of PTN, where Δ*A* denotes the difference in absorbance in the absence and presence of HP-β-CD:

(1) $$R=\frac{\left[PTN\right]}{\left[PTN\right]+\left[HP\text{-}\beta \text{-}CD\right]}$$

### 2.5. Phase Solubility Study

A phase solubility study was performed in water at 25 °C according to the method described by Higuchi and Connors [35]. A known excess of PTN was added to vials containing different concentrations of HP-β-CD (0.28 mM-35.7 mM), and the suspensions were stirred at 25 °C for 48 h on a magnetic stirrer at 200 rpm (IKA RT 15, IKA-Werke, Staufen, Germany). Uncomplexed PTN was removed by centrifugation at 16,800× *g* for 15 min (Centrifuge 5418, Eppendorf, Hamburg, Germany), and PTN concentration was measured spectrophotometrically at λ = 434 nm (UV mini-1240, Shimadzu, Kyōto, Japan). The phase solubility graph was constructed by plotting PTN concentration (mM) against HP-β-CD concentration, and the apparent stability constant (K_S_) was calculated according to the following equation.

(2) $$K_{S}=\frac{Slope}{\left[S_{0}\left(1-Slope\right)\right]}$$

*S*_0_ is the intrinsic solubility of PTN in water as measured in our study to be 0.17 µg/mL.

### 2.6. Preparation of PTN/HP-β-CD Complexes and Physical Mixture

PTN/HP-β-CD inclusion complexes were prepared by the freeze-drying method [10,34]. Briefly, PTN and HP-β-CD (at a molar rate of 1:1) were dissolved in ethanol and ultrapure water, respectively. PTN was added stepwise to the HP-β-CD aqueous solution placed on a magnetic stirrer at 200 rpm and 25 °C for 48 h (IKA RT 15, IKA-Werke, Staufen, Germany). Insoluble PTN was removed by centrifugation at 16,800× *g* for 15 min and filtration (0.45 µm nylon filter, Pall Corporation, New York, USA). The obtained solution was then lyophilized in a freeze-dryer (Christ Alpha 1-4 LSC, Martin Christ Gefriertrocknungsanlagen, Osterode am Harz, Germany). The physical mixture was prepared by mixing PTN and HP-β-CD (in a 1:1 molar ratio) in a porcelain mortar with grinding for 15 min and then stored in a desiccator until further analysis.

### 2.7. Characterization of PTN/HP-β-CD Complexes

#### 2.7.1. ^1^H NMR Spectroscopy and 2D ROESY

^1^H NMR spectroscopic measurements were recorded by an NMR spectrometer equipped with an auto-tune sample head (JEOL ECX-400 Nuclear Magnetic Resonance Instrument, JEOL, Akishima, Japan). Samples were prepared by dissolving an equivalent amount of free and complexed PTN in a suitable NMR solvent (DMSO-d6). Samples were then transferred into NMR tubes and assessed through several scanning cycles fixed to a minimum of 64 scans. The results were processed by MNOVA software (version 14.2.1, Mestrelab Research S.L., Santiago de Compostela, Spain).

#### 2.7.2. FT-IR Analysis

The FT-IR measurements were performed by a Brucker α-alpha FT-IR instrument (Bruker Optic, Ettlingen, Germany) with an attenuated total internal reflectance diamond crystal. The neat solid samples were placed directly on the diamond crystal, after which the adjustable pressure arm was positioned over the sample to press it gently. FT-IR spectra were recorded in the transmission mode from 4000 to 400 cm^−1^ [36].

#### 2.7.3. Powder X-ray Diffraction (PXRD)

The formation of PTN/HP-β-CD complexes was confirmed by XRD, determining its crystallinity after inclusion. XRD patterns of PTN/HP-β-CD complexes, the corresponding physical mixture, and the individual solid components were recorded with CuK α radiation (λ = 1.7903 Å) at a voltage of 40 kV and 35 mA current (X’Pert Pro MDP X-ray powder diffractometer, PANalytical, Almelo, Netherlands). Samples were scanned at room temperature from 2θ = 10° to 2θ = 60° with a step of 0.03°/min.

#### 2.7.4. Scanning Electron Microscopy (SEM)

The surface morphology of the PTN/HP-β-CD inclusion complexes was investigated by SEM (Hitachi S-510, Hitachi-High Technologies Europe, Krefeld, Germany) equipped with a secondary electron detector. Briefly, the individual components (pure PTN, HP-β-CD), their physical mixture, and PTN/HP-β-CD inclusion complexes were mounted on an aluminum pin stub by conductive double-sided adhesive carbon tabs. The powders were sputter-coated thrice with a thin layer of gold (10 mA for 1 min) in an Edwards S150 Sputter Coater (Edwards Vacuum, Crawley, UK). The samples were visualized at an acceleration voltage of 10 kV.

#### 2.7.5. Differential Scanning Calorimetry (DSC)

The thermal behavior of the obtained complex and its pure substances were assessed by DSC measurements (DSC-7, Perkin Elmer, Rodgau, Germany). Briefly, accurately weighted amounts of the solid materials were filled into aluminum pans and heated over the temperature range 20–300 °C at a rate of 10 °C min^−1^ under a nitrogen purge for cooling. In parallel, an empty pan sealed in the same way was served as a reference.

#### 2.7.6. UV/Vis Absorption Spectroscopy

The absorption spectrum of PTN/HP-β-CD inclusion complexes (80 µg/mL) was obtained from λ = 200 to 700 nm by a UV/Vis spectrophotometer (Multiskan GO, Thermo Scientific, Waltham, MA, USA) and compared with that of free PTN in ethanol. The absorbance spectra were recorded using an aqueous solution of HP-β-CD or pure ethanol as background references for PTN/HP-β-CD complexes and free PTN, respectively.

#### 2.7.7. Singlet Oxygen Quantum Yield

The photodynamic activity of PTN/HP-β-CD complexes was evaluated by analyzing the singlet oxygen generation (^1^O_2_) using uric acid (UA), and as previously mentioned, using rose bengal (RB) as a standard photosensitizer with a reported singlet oxygen quantum yield of 0.75 in water [19]. Briefly, uric acid (100 µM) was mixed with PTN (10 µg/mL) and irradiated 5 times for 1 min by a blue LED (λ_irr_ = 457 nm, irradiance = 220.2 W/m^2^, Lumundus, Eisenach, Germany). The absorption spectra of UA were recorded after each irradiation procedure by a UV-Vis spectrophotometer (Multiskan GO, Thermo Scientific, Waltham, MA, USA). The normalized UA absorbance at λ = 296 nm was plotted versus the irradiation time (in seconds), and the decomposition rate constant of uric acid was calculated to quantify the singlet oxygen quantum yield using the following equation.

(3) $$\Phi {{[}^{1}O_{2}]}_{PTN}=\Phi {{[}^{1}O_{2}]}_{RB}\;\frac{K_{PTN}\;F_{RB}}{\begin{array}{c} K_{RB}\;F_{PTN} \end{array}}$$

$\Phi {{[}^{1}O_{2}]}_{PTN}$ and $\Phi {{[}^{1}O_{2}]}_{RB}$ are the singlet oxygen quantum yields of PTN and RB, respectively. k_PTN_ and k_RB_ are the rate constants of uric acid degradation by PTN and RB, respectively. F is the absorption correction factor given by F = 1 − 10^–OD^ (OD at the irradiation wavelength).

#### 2.7.8. Photostability of Inclusion Complexes

The effect of complexation on photodegradation of PTN was studied, and the degradation profile was compared with that of free PTN in ethanol. Solutions of either PTN/HP-β-CD complexes in water or free PTN in ethanol (100 µg/mL) were irradiated by an LED device (λ_irr_ = 457 nm, irradiance = 220.2 W/m^2^) at 5 min interval with a total irradiation time of 30 min. After each irradiation time, the absorbance spectra were measured at λ_max_ = 434 nm (Multiskan GO, Thermo Scientific, Waltham, MA, USA). The absorbance spectra were normalized for better comparison, and PTN’s remaining percentage was calculated and compared with free PTN [37].

### 2.8. Bacterial Viability Assay

The antibacterial activity was determined by incubating the formulations with the bacterial suspensions and irradiating the samples. Both microorganisms (*S. saprophyticus* and *E. coli*) were treated equally, and similar bacterial densities were used. The overnight cultures were diluted to an optical density (OD_600_) of 0.025 measured by a spectrophotometer (Shimadzu UV mini-1240, Kyōto, Japan). These suspensions were placed in an orbital shaker (Compact Shaker KS 15 A, equipped with Incubator Hood TH 15, Edmund Bühler) set at 300 rpm and 37 °C. The growth of the bacterial suspensions was stopped by placing them on ice at an OD_600_ of 0.4. A total of 150 uL of each bacterial suspension was incubated with an equal volume of PTN/HP-β-CD complexes (containing 200 µM PTN) in 12-well cell culture plates (TC plate standard, Sarstedt, Nümbrecht, Germany) for 30 min at 37 °C and 100 rpm, so that the final PTN concentration was set to 100 µM. These solutions were irradiated with blue-LED (λ_irr_ = 457 nm) for 30 min at a radiant exposure of 39.6 J/cm^2^. After irradiation, the suspensions were serially diluted with MHB and plated onto Mueller Hinton II Agar plates (BD, Heidelberg, Germany). After incubating the plates for 18 h at 37 °C and 90% relative humidity, the viable colonies were counted and the colony-forming units per milliliter were calculated [CFU/mL]. Filter-sterilized phosphate-buffered saline (PBS) (pH 7.4) was used as a control. The experiments were performed in triplicates.

### 2.9. Statistical Analysis

Unless otherwise stated, all experiments were performed in triplicates, and the results are expressed as means ± standard deviations. A two-tailed Student’s t-test was performed to identify statistically significant differences. Probability values of *p* < 0.05 were considered significant.

## 3. Results and Discussion

### 3.1. Stoichiometry: Job’s Plot

**Figure 1:**
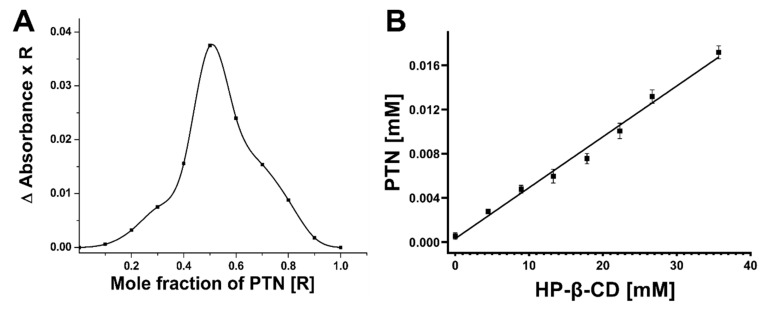
(**A**) Job’s plot for different mole fractions of PTN (R). (**B**) Phase solubility diagram of PTN/HP-β-CD complex system at 25 °C. The concentration of HP-β-CD was in the range of 0–35 mM. All measurements were performed in triplicate, and the values were expressed as means ± SDs (*n* = 3).

Job’s method was employed to determine the stoichiometry of PTN and HP-β-CD, which was indicated by a maximum on Job’s plot at a specific molar ratio. As shown in Figure 1A, (ΔAbs x R) is greatest at a mole fraction of 0.5, indicating that the stoichiometry between PTN and HP-β-CD is 1:1, where a single PTN molecule can be included in the cavity of one HP-β-CD molecule. Qiu et al. dealt with the complexation of anthraquinone, emodin, in HP-β-CD [34], and the authors presented a similar profile with 1:1 stoichiometry between emodin and HP-β-CD.

### 3.2. Phase Solubility Study

The phase solubility was conducted by evaluating the change in PTN solubility as a function of HP-β-CD concentration to determine the stoichiometric ratio and stability constant of PTN in HP-β-CD. The inclusion complex formation was visually observed by the typical yellow color of PTN, indicating its solubilization in HP-β-CD solution. As depicted in Figure 1B, PTN solubility increased proportionally with an increase in HP-β-CD concentration, resulting in a correlation coefficient of 0.975 over the studied concentration range. PTN solubility in water rose considerably from 0.00059 mM in the absence of HP-β-CD to 0.017 mM in the presence of 35 mM HP-β-CD because of the inclusion of PTN within the hydrophobic cavity of HP-β-CD. This host-guest system is a typical A_L_ type revealing soluble complex formation according to Higuchi and Connors [35] and showing a 1:1 stoichiometry between PTN and HP-β-CD. The slope in Figure 1B is below unity, implying the formation of an inclusion complex at a molar ratio of 1:1 consistent with Job’s plot. The stability constant is K_S_ = 733.46 M^−1^, and according to the literature, it is considered optimal in the range of 50 to 2000 M^−1^ [34]. Smaller values suggest poor interactions between the guest and the CD, whereas higher values indicate difficult complex dissociation, leading to incomplete guest release from the inclusion complex. As a result, a 1:1 molar ratio of PTN and HP-β-CD was employed for the inclusion complex formation for further characterization.

### 3.3. Characterization of PTN/HP-β-CD Complexes

#### 3.3.1. ^1^H NMR Spectroscopy and 2D ROESY

**Figure 2:**
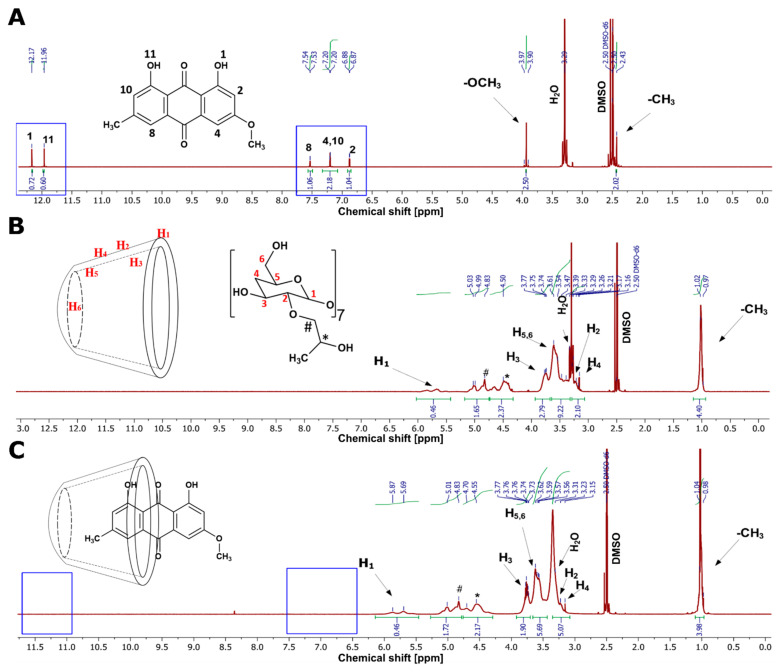
^1^H NMR spectra of (**A**) PTN, (**B**) HP-β-CD, and (**C**) the PTN/HP-β-CD complexes in DMSO-d_6_.

The ^1^H NMR investigations for free and complexed PTN were performed to explore the possible inclusion mode of the PTN/HP-β-CD complexes [38]. Accordingly, we compared the NMR spectra of free PTN, pure HP-β-CD, and the PTN/HP-β-CD complexes. As illustrated in Figure 2A, pure PTN in DMSO-d6 showed peaks corresponding to the methyl and methoxy protons at 2.4 and 3.91 ppm, respectively. In addition, the aromatic ring protons exhibited their corresponding peaks at 6.8, 7.1, and 7.5 ppm. The intense peaks at 11.9 and 12.1 ppm corresponded to the phenolic protons on each side of the anthraquinone structure [39]. Figure 2B indicated the prominent peaks of pure HP-β-CD in DMSO-d6, showing the primary chemical shifts for H-1 to H-6 protons between 3 and 6 ppm except for the methyl protons that appeared at 1 ppm. The inclusion of PTN into HP-β-CD resulted in the disappearance of the PTN’s aromatic and hydroxyl protons between 6.5 and 12 ppm, as annotated in Figure 2C. Moreover, the methyl and methoxy protons entirely vanished at 2.4 and 3.9 ppm.

**Figure 3:**
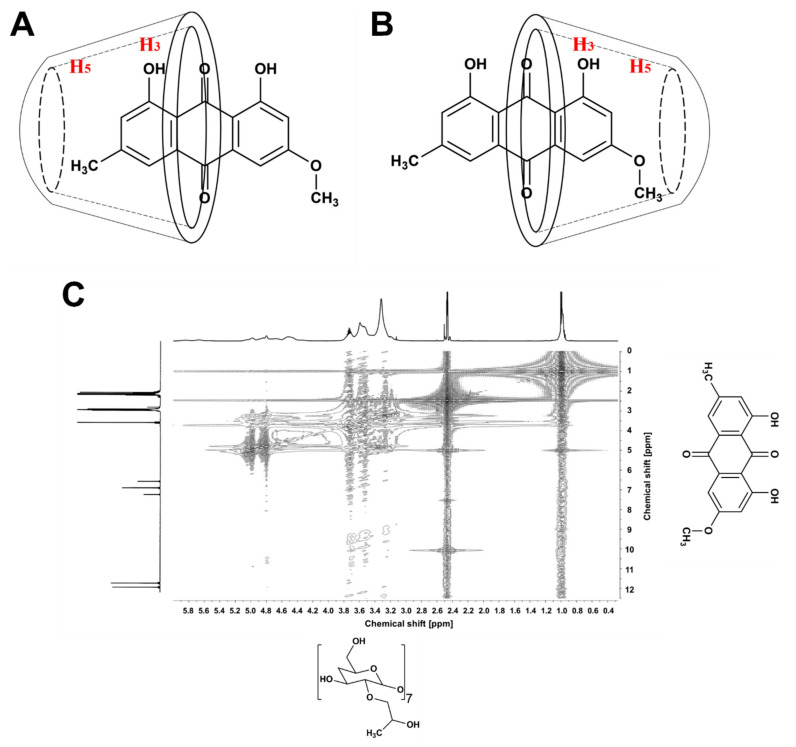
The possible penetration patterns of PTN through the HP-β-CD cavity by either the methyl (**A**) or the methoxy (**B**) substituted rings. (**C**) ROESY spectrum of the PTN/HP-β-CD complexes in DMSO-d6.

For further explanation of the inclusion pattern, the chemical shifts in the presence and absence of PTN were recorded (Table 1). The inclusion of PTN into the HP-β-CD had a negligible influence on the H-4, H-5, and H-6 protons (0.01 ppm). In contrast, the values for H-2 and H-3 indicated a slightly significant change (0.02–0.05 ppm), which might be brought about by the spatial interaction of these protons with the hydroxyl protons of PTN. It is worth noting that the H-3 and H-5 protons are on the interior side of the CD cavity, with the H-3 near the wider opening and the H-5 protons near the narrower side of HP-β-CD [40]. Because of the minor change in the chemical shift of the H-5 (about 0.01 ppm) compared to the more considerable change in the H-3 (about 0.02 ppm), we could presume that PTN might interact with the HP-β-CD through its wider side. According to these findings, PTN is suspected of penetrating the HP-β-CD cavity by its methyl or methoxy substituted rings. The disappearance of their corresponding protons supported this after inclusion with HP-β-CD. Therefore, we hypothesized that PTN could have two inclusion possibilities, as illustrated in Figure 3A,B.

Furthermore, the 2D ROESY of the PTN/HP-β-CD complexes was obtained to attain more conformational details of the spatial configuration of the complexed PTN [41]. The ROESY spectrum of the PTN/HP-β-CD complexes (Figure 3C) showed a relative correlation between the hydroxyl protons of PTN and the H-3 protons of the CD molecule, which is likely driven by hydrogen bonding forces. Moreover, the aromatic protons of PTN at positions 2, 4, 8, and 10 had a spatial correlation with the H-2 and H-5 protons of HP-β-CD, which explains the significant proton shifting of the H-2 (0.05 ppm) and strengthens the hypothesis that PTN was introduced through the broader side of the CD cavity.

#### 3.3.2. FT-IR Analysis

**Figure 4:**
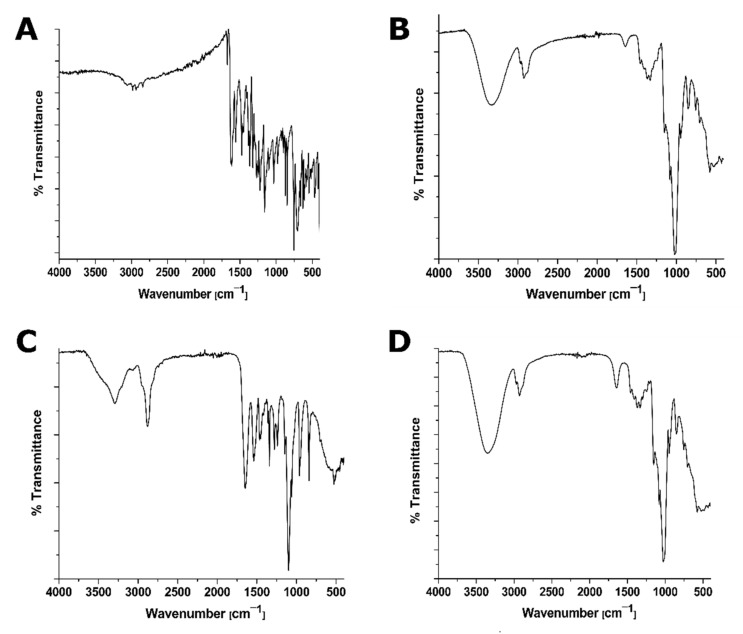
FT-IR spectra of (**A**) PTN, (**B**) HP-β-CD, (**C**) physical mixture (1:1 molar ratio of PTN and HP-β-CD), and (**D**) PTN/HP-β-CD complexes over the wavenumber range 4000–400 cm^−1^.

The possible interactions between PTN and HP-β-CD in the solid-state were assessed by comparing the FT-IR spectra of pure PTN, HP-β-CD, their physical mixture, and PTN/HP-β-CD complexes. The FT-IR spectrum of pure PTN (Figure 4A) shows many absorption bands: at 2936 cm^−1^ (CH_3_ asymmetric), 2844 cm^−1^ (CH_3_ symmetric), 1674–1613 cm^−1^ (C=O free and conjugated), 1557 cm^−1^ (C=C aromatic), and 1383–1364 cm^−1^ (C–O phenyl) as previously reported [39]. The pure HP-β-CD spectrum (Figure 4B) showed a broad band at 3331 cm^−1^ corresponding to OH stretching vibrations of various hydroxyl groups. The absorption bands at 2922 cm^−1^, 1640 cm^−1^, and 1149 cm^−1^ are also related to CH_2_ stretching vibrations, O-H bending vibrations, and C-O-C stretching vibrations, respectively. The absorption bands of the valence vibrations of the C–O bonds in the ether and hydroxyl groups of HP-β-CD (1082 and 1035 cm^−1^) were noticed as previously reported [42]. Figure 4C depicts that the physical mixture spectrum was equivalent to a simple combination of PTN and HP-β-CD. However, the characteristic carbonyl peaks of pure PTN were present in the physical mixture but absent in PTN/HP-β-CD complexes (Figure 4D). Also, there is neither a band at 1557 cm^−1^ nor at 1383–1364 cm^−1^ assigned to C=C aromatic and C–O phenyl, respectively. The band of the valence vibration of the O–H bond in PTN/HP-β-CD complexes was shifted to 3353 cm^−1,^ and this was related to water release upon host-guest interaction as observed previously in the literature [42]. Overall, the inclusion complex did not display any new IR peaks signifying that no chemical bonds are formed with the obtained complex.

#### 3.3.3. Powder X-ray Diffraction (PXRD)

**Figure 5:**
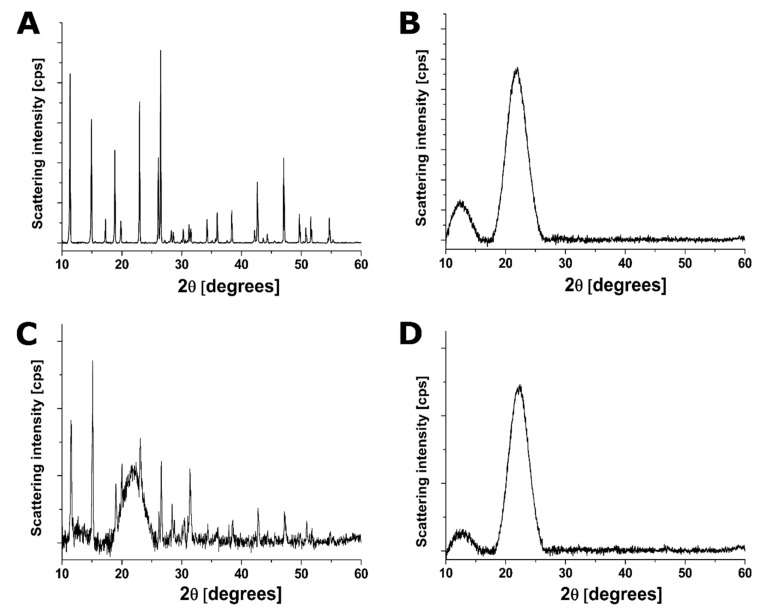
Powder X-ray diffraction patterns of (**A**) PTN, (**B**) HP-β-CD, (**C**) physical mixture (1:1 molar ratio of PTN and HP-β-CD), and (**D**) PTN/HP-β-CD complexes. Scanning angle of 2θ = 10–60; step width of 0.03°/min.

PXRD was used to identify any change in the crystallinity of PTN upon complexation. The PXRD pattern of pure PTN (Figure 5A) displayed well-resolved characteristic diffraction peaks at different 2*θ*, revealing its crystalline character. The position of peaks corresponds to what has previously been described in the literature [43]. In the case of HP-β-CD (Figure 5B), a diffuse pattern without any sharp peaks was obtained, indicating its amorphous nature. The physical mixture (Figure 5C) exhibited almost all of the distinctive peaks of PTN and HP-β-CD but with lower intensity confirming the presence of both components as isolated species while preserving the crystalline nature of PTN.

On the other hand, the diffractogram of PTN/HP-β-CD complexes (Figure 5D) exhibited the broad peak of pure HP-β-CD with the absence of peaks assigned to pure PTN, which may be due to the presence of the drug in a molecularly dispersed form in HP-β-CD. According to the literature, the amorphous structure is attributed in part to the HP-β-CD structure and in part to the lyophilization step during preparation [44]. These results confirm those obtained by FT-IR, which imply the disappearance of drug crystallinity in the obtained complex.

#### 3.3.4. Scanning Electron Microscopy (SEM)

**Figure 6:**
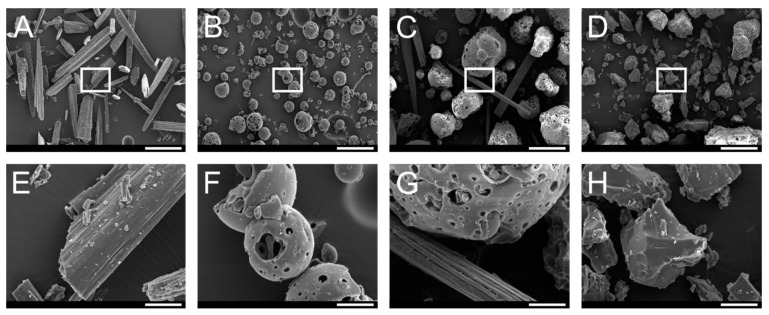
SEM micrographs recorded at an acceleration voltage of 10 kV of (**A**) PTN, (**B**) HP-β-CD, (**C**) physical mixture (1:1 molar ratio of PTN and HP-β-CD), and (**D**) PTN/HP-β-CD complexes with the respective magnification (**E**–**H**) corresponding to the white rectangle. Scale bars represent 200 µm in (**A**–**D**) and 30 µm in (**E**–**H**).

SEM was used to demonstrate the morphological changes in PTN upon formation of PTN/HP-β-CD complexes, and the results are presented in Figure 6. Pure PTN appeared as irregularly sized needle-shaped crystals (Figure 6A), while pure HP-β-CD existed as spherical crystals, well separated from each other with a porous surface (Figure 6B). The shape of the physical mixture and the inclusion complex was completely different. SEM pictures of the physical mixture indicated no change in the crystal state regarding the original morphology of individual components. The needle-shaped PTN crystals are distributed between HP-β-CD crystals (Figure 6C). However, PTN/HP-β-CD complexes appeared as roughly rectangular homogeneous particles without the spherical shape of HP-β-CD or the needle shape of PTN. This morphological alteration to a single solid phase can indicate a successful formation of PTN/HP-β-CD complexes and is consistent with the data obtained in the FT-IR and XRPD studies.

#### 3.3.5. Differential Scanning Calorimetry (DSC)

**Figure 7:**
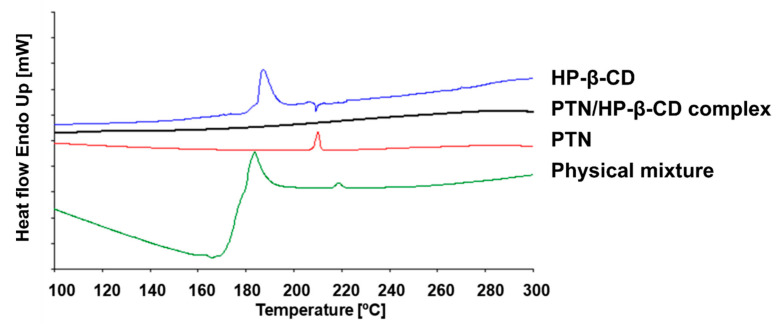
DSC curves of HP-β-CD, PTN, physical mixture (1:1 molar ratio of PTN and HP-β-CD), and PTN/HP-β-CD complexes. The heating rate was 10 °C min^−1^. The thermograms were adjusted for better visualization.

DSC is widely used to characterize the inclusion of active moieties in the CD cavity by a shift or disappearance in the melting point of the guest molecules [45]. Figure 7 displays that pure PTN exhibits a sharp endothermic peak at 210 °C corresponding to its melting point, indicating its crystalline nature, which is well-matched with previous reports [39,46]. As reported before, the pure HP-β-CD showed a broad peak at 187 °C because of water loss from the cyclodextrin cavity at higher temperatures [47]. As clearly evidenced in Figure 7, the physical mixture thermogram was quite different from that of the pure components. In the physical mixture, the PTN peak was reduced in intensity and shifted to a higher temperature (218 °C), while HP-β-CD shifted to a lower temperature (183 °C) in comparison to the pure HP-β-CD (187 °C). This may be due to interactions during the DSC run, at least within the ~100–180 °C temperature range, and agrees with a previous study attributing these slight peak shifts to the weak interaction between the cyclodextrin and drug during the physical mixture preparation [48].

On the contrary, Figure 7 depicts a notable difference in the thermal profile of PTN/HP-β-CD complexes in comparison to the parent material and their physical mixture. The endothermic peak of PTN melting was no longer visible, revealing the amorphous state of the drug because of an interaction between PTN and HP-β-CD as a result of guest–host inclusion complex formation. The absence of drug and cyclodextrin peaks in the complex thermogram indicated successful inclusion complex formation. Moreover, the dehydration peak of HP-β-CD appeared only in the pure HP-β-CD and in the physical mixture thermograms but was unseen in those of PTN/HP-β-CD complexes, confirming the complex formation. The plausible explanation of this phenomenon may be that the cyclodextrin cavity was occupied with the hydrophobic drug and no space was available for water molecules [48].

#### 3.3.6. UV/Vis Absorption Spectroscopy

**Figure 8:**
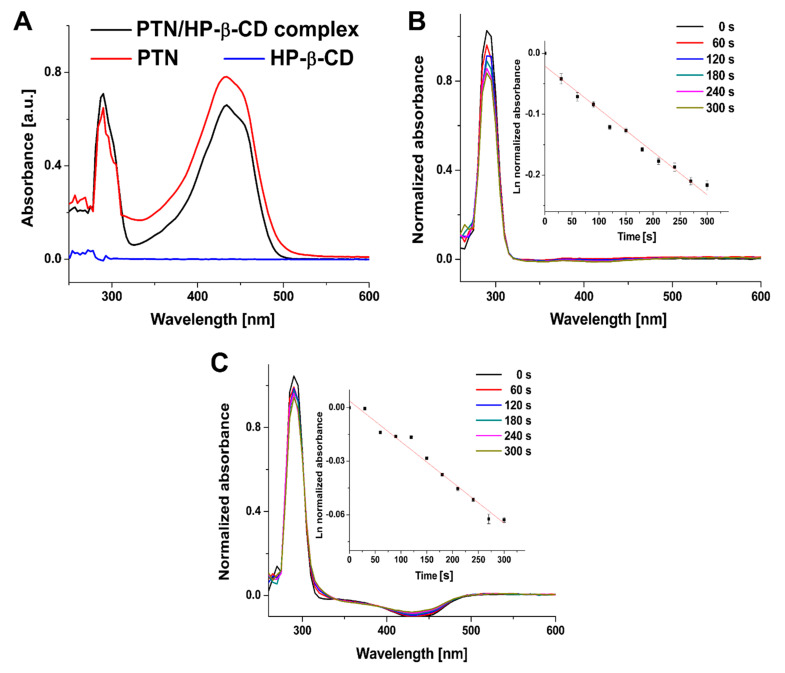
(**A**) UV/Vis spectra of PTN in ethanol (80 µg/mL), HP-β-CD in water (0.5 mg/mL) and PTN/HP-β-CD inclusion complexes in water (80 µg/mL). (**B**,**C**) Absorption spectra of uric acid upon irradiation for different times in the presence of (**B**) free PTN and (**C**) PTN/HP-β-CD complexes. The insets represent the plot of Ln normalized absorbance of uric acid at λ = 296 nm versus irradiation time in s (λ_irr_ = 457 nm, irradiance = 220.2 W/m^2^). All measurements were performed in triplicate, and the values were expressed as means ± SDs (*n* = 3).

Figure 8A shows that HP-β-CD has no absorption peak within the recorded spectrum as it does not have any double bond (π-electrons) to absorb UV energy, as previously reported [49]. As depicted in Figure 8A, the UV-Vis absorption spectra of free and complexed PTN have two characteristic absorption peaks at λ_max_ = 290 nm and 434 nm, respectively, similar to those reported before [17]. The UV-Vis absorption spectra of free PTN and PTN/HP-β-CD complexes were comparable along the scanned wavelength. The peak position is similar to that of free PTN in ethanol, revealing that the complexation had no significant effect on the PTN absorption spectrum. Moreover, it indicates that the solubility of PTN in water is enhanced without aggregations. However, a slightly hypochromic shift was observed in PTN/HP-β-CD complexes, which could be attributed to the more hydrophilic microenvironment surrounding PTN [50]. Similar behavior was reported by Cannavà et al. [51], who studied the effect of sulfobutyl ether β-cyclodextrin on idebenone and reported a significant hypochromic shift because of the complexation within the cyclodextrin cavity [51]. The absorption spectrum of nifedipine was also almost identical to the free form but with lower absorbance intensity at some wavelengths [52].

#### 3.3.7. Singlet Oxygen Quantum Yield

Since the phototoxicity of the photosensitizer involves the production of cytotoxic species such as singlet oxygen, it is useful to investigate the singlet oxygen generation efficiency. This can be performed by monitoring the decay curves of UA absorbance as a function of irradiation time. Following the established measuring procedure [19], the decomposition of UA upon exposure to generated singlet oxygen was employed to measure singlet oxygen generation. UA was selected because it does not absorb light in the blue region, specifically at the PTN irradiation wavelength. A similar irradiation experiment was also performed in parallel as a negative control, where UA solution was irradiated with a blue LED. In the absence of PTN, no photobleaching of UA was observed after irradiation (data not shown), and therefore, any loss in UA absorbance upon irradiation is due to the photodynamic activity of PTN. Figure 8B,C depicts that the decrease in UA absorbance is a function of light exposure time, indicating the irradiation time-dependent singlet oxygen production, with the lowest UA absorbance at 5 min irradiation time. A good linear relation between Ln normalized absorbance of UA at λ = 296 nm and the irradiation time denotes that the decay kinetics is first order.

A comparison of UA decays in Figure 8B revealed that free PTN induced significantly faster UA degradation than observed in the case of complexed PTN (Figure 8C). The rate constants for UA decomposition were 7.04 × 10^−4^ and 2.28 × 10^−4^ s^−1^, while the singlet oxygen quantum yields were 0.49 and 0.35 for free and complexed PTN, respectively. This decrease in singlet oxygen generation upon complexation with HP-β-CD may be due to the photosensitizers’ environment affecting the quantum yield. It is well-established that singlet oxygen has a longer lifetime in organic solvents than in water, where fast quenching of singlet oxygen between water molecules reduces the singlet oxygen lifetime [53]. Moreover, PTN/HP-β-CD complexes may be more stable and less susceptible to photodegradation than ethanolic PTN solutions. According to the literature, cyclodextrin complexation changes the photochemical characteristics of the guest molecules and has been long used to protect drugs against photodegradation [54]. Previous research suggests that β-cyclodextrin complexation has a negligible effect on the UV spectra, but it causes significant changes in the emissions spectra and fluorescence quantum yields as it usually impacts the ground and the excited states [50]. These results further confirm that in water, HP-β-CD maintains the PTN molecules mainly as monomeric species, which are the photoactive form for ROS generation.

#### 3.3.8. Photostability of Inclusion Complex

**Figure 9:**
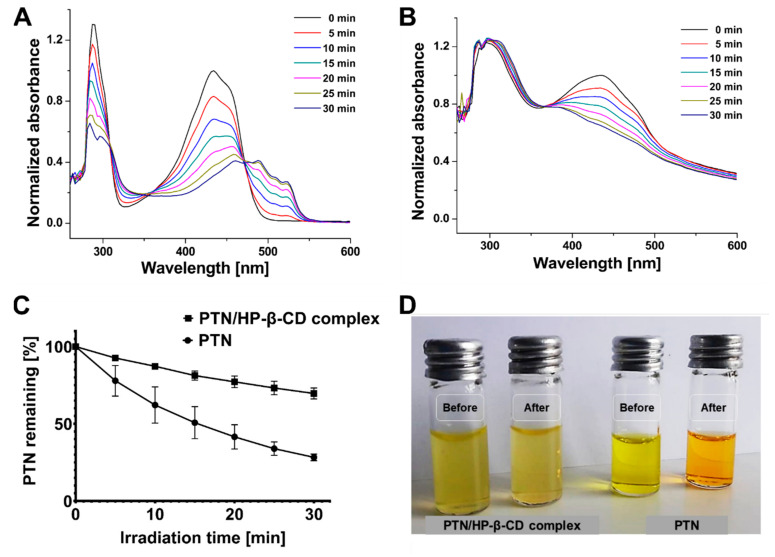
Effect of HP-β-CD on the photodegradation of PTN after blue LED irradiation at different time intervals (λ_irr_ = 457 nm, irradiance = 220.2 W/m^2^). (**A**) The absorption spectra of PTN in ethanol and (**B**) the absorption spectra of PTN/HP-β-CD complexes in ultrapure water. (**C**) Decrease in the PTN content of free and complexed PTN at different irradiation intervals with a blue LED. (**D**) Photomicrograph of PTN/HP-β-CD complexes and PTN before and after 30 min irradiation. All measurements were performed in triplicate, and the values were expressed as means ± SDs (*n* = 3).

Considering the practical use of PTN/HP-β-CD complexes in aPDT, it is better to investigate the influence of irradiation time on the degradation of free and complexed PTN. Figure 9A,B show that the decrease in absorbance measured at λ_max_ = 434 nm is light-dependent, with the lowest absorbance detected after 30 min irradiation. This may be due to several photoactivations of the photosensitizer with successive irradiation, leading to photodecomposition or photobleaching. As shown in Figure 9C, less than 28% of free PTN remained after 30 min irradiation compared to about 70% in the case of the complexed form, indicating a better photostability of HP-β-CD. Figure 9D presents that complexed PTN could retain its characteristic yellow color even after 30 min irradiation. The slight color change of free PTN to yellowish-orange is most probably due to the formation of photodegradation products evidenced by a decrease in the peak intensity at 434 and 287 nm and a simultaneous increase of the new small peak at 488 nm, which appeared only after irradiation with the highest intensity after 30 min (Figure 9A). This photostability can be beneficial in PDT since PS molecules are still available for further photoactivation leading to more ROS generation with additional irradiation.

Additionally, the photobleaching of free PTN could limit the irradiation time and reduce its photosensitizing efficacy, especially when a longer irradiation time is required. This is consistent with a previous study, where HP-β-CD complexed cilnidipine exhibited lower photodegradation than free cilnidipine in ethanol, and the authors related this to the protective effect conferred by the host-guest system [55]. This effect was also reported before for curcumin after complexation in cyclodextrin [56]. According to the literature [57], the host-guest structure can reduce the hydrolytic or photolytic degradation of a drug in inclusion complexes by providing a molecular shield against reactive substances. This shielding effect can presumably result in lower availability and interaction of complexed PTN molecules with the light source, leading to lower singlet oxygen generation as stated before compared to the free PTN.

### 3.4. Bacterial Viability Assay

**Figure 10:**
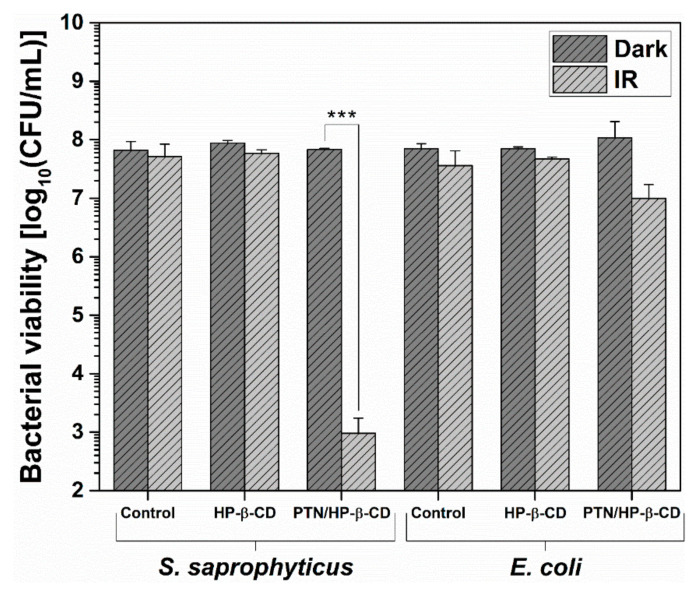
Bacterial viability of *Staphylococcus saprophyticus* subsp. *bovis* (*S. saprophyticus*) and *Escherichia coli* DH5 alpha (*E. coli*) treated with PTN/HP-β-CD complexes (100 µM PTN) or only with HP-β-CD for 30 min at 37 °C and then irradiated (IR) with a blue LED (λ_irr_ = 457 nm, 39.6 J/cm^2^) or kept in the dark. Control represents bacterial suspension treated with filter-sterilized phosphate-buffered saline (PBS) (pH 7.4). The results are expressed as means ± SDs (*n* = 3). Statistical differences are denoted as “***” *p* < 0.001.

In this study, antibacterial activity was examined by irradiating bacteria treated with the prepared PTN/HP-β-CD complexes, and the results were presented in Figure 10. For the used concentration (100 µM PTN), no significant dark toxicity could be observed toward either microorganism when comparing the non-irradiated control sample and the non-irradiated formulation. In addition, the effect of the light source was negligible as it did not influence bacterial viability as seen in the irradiated control sample [9,58,59]. Furthermore, the unirradiated and irradiated HP-β-CD also showed no significant change in bacterial viability. In contrast, the irradiated PTN/HP-β-CD complexes significantly reduced the bacterial viability of the gram-positive *S. saprophyticus* by > 4.8 log. Therefore, it can be assumed that the antibacterial effect originated exclusively from the irradiated PTN/HP-β-CD complexes. According to the American Society for Microbiology, a bacterial reduction of 99.9986% qualifies the formulation as antibacterial [60]. The viability of the gram-negative *E. coli* was reduced by > 1.0 log after incubation with PTN/HP-β-CD complexes and irradiation. As described in the literature, the antibacterial effect of PTN is lower with gram-negative germs, and higher concentrations are needed for the same effect [14]. In addition, the affinity of the cyclodextrin complex to gram-negative bacteria can be slightly reduced because of their unique cellular structure. The difference in the cell wall structure between gram-negative and gram-positive bacteria may account for their different response to aPDT. The accumulation of PSs inside gram-negative bacteria is limited because of two mechanisms. Firstly, the lipid-rich membrane bilayer enclosing the cell wall reduces the inward penetration of PTN/HP-β-CD complexes into gram-negative bacteria [5]. Secondly, gram-negative bacteria widely use porins and efflux pumps to regulate nutrient and toxin influx and efflux, and hence they act as natural resistance mechanisms [5]. However, compared with antibacterial studies on pure PTN, the concentration used here is reduced almost tenfold. Comini et al. showed no dark toxicity of PTN against *E. coli* up to 320 µg/mL, and an antibacterial activity of irradiated PTN only above a concentration of 250 µg/mL [17]. This underlines the superiority of the cyclodextrin complex at a used PTN concentration of just 28.45 µg/mL. Overall, the complexation of PTN not only improved the water solubility and photostability of PTN but also increased the antibacterial effect, enabling the therapeutic application of PTN.

## 4. Conclusions

The efficacy of parietin (PTN) as a natural photosensitizer is poor because of its hydrophobic structure and π–π stacking, which consequently results in aggregation-induced quenched fluorescence, limited ROS production, and a diminished photodynamic effect. In this study, HP-β-CD was employed to enhance the water solubility of PTN and decrease its aggregation in aqueous vehicles. The water solubility of PTN was enhanced up to 28-fold after complexation with HP-β-CD. Various characterization methods confirmed that PTN was adequately included in the cyclodextrin cage. Even though PTN/HP-β-CD complexes have a polar environment, they are still photoactive because HP-β-CD provides a protecting hydrophobic cavity for PTN, resulting in a shielding effect from water molecules. This can be a critical factor in maintaining its photodynamic activity, as confirmed by uric acid degradation due to singlet oxygen generation. Additionally, the complexation was shown to slow down the photodegradation of PTN. Although PTN is reported to have a dark antimicrobial effect, it is presumed that aPDT could considerably reduce the required dose to give a comparable effect and lower toxic effects. In the long-term prospect, the limited efficacy of PTN/HP-β-CD against gram-negative bacteria may be overcome by the delivery of PTN in cationic formulations.

## Figures and Tables

**Table 1:** Selected chemical shifts (δ in ppm) of HP-β-CD and PTN/HP-β-CD complexes in DMSO-d6.

Protons	δ (ppm)	
	HP-β-CD	PTN/HP-β-CD Complexes
**H-1**	5.64	5.66
**H-2**	3.27	3.32
**H-3**	3.72	3.74
**H-4**	3.13	3.12
**H-5**	3.54	3.53
**H-6**	3.58	3.59
**CH_3_**	1.00	1.00

## Data Availability

The data presented in this study are available on request from the corresponding author.
